# Skeletal and dental characteristics in subjects with ankyloglossia

**DOI:** 10.1186/2196-1042-14-44

**Published:** 2013-11-07

**Authors:** Bhadrinath Srinivasan, Arun B Chitharanjan

**Affiliations:** Department of Orthodontics, Sri Ramachandra University, Porur, Chennai, 600116 India

## Abstract

**Background:**

The role of ankyloglossia in etiology of malocclusion is not much discussed over the years. The aim of the present study was to assess the skeletal and dental characteristics in subjects with ankyloglossia.

**Methods:**

Fifty-seven subjects diagnosed with ankyloglossia (group 1) were compared with 60 subjects (group 2) without ankyloglossia, who had class I skeletal base. Ankyloglossia was diagnosed and graded (mild, moderate, severe and very severe) using Kortlow's method. SNA, SNB, ANB, Go-Gn-SN angle, FMA, maxillary and mandibular intercanine widths and intermolar widths, tooth size-arch length discrepancy in maxillary and mandibular arches and overbite were measured. Independent *t* test was used to compare the mean parameters between the two groups. Analysis of variance and Tukey honestly significant difference were used to compare mean parameters among various grades of ankyloglossia.

**Results:**

Majority of group 1 subjects belonged to class I skeletal base followed by class II and class III skeletal bases. Moderate ankyloglossia was most common in group 1. The mean maxillary and mandibular intercanine widths and maxillary intermolar width were statistically significant in independent *t* test (*P* < 0.01) and reduced in group 1. In ANOVA followed by Tukey HS, the Go-Gn-SN angle and overbite were statistically significant among different grades of ankyloglossia (*P* < 0.05).

**Conclusions:**

Subjects with ankyloglossia had reduced maxillary and mandibular intercanine widths and reduced maxillary intermolar width. The mandibular plane angle and overbite were altered with severity of ankyloglossia.

## Background

Frenum is a fold of tissue inside the oral cavity which connects structures like the lip, tongue and buccal musculature to the alveolar bone. The frenums in the oral cavity include the maxillary midline frenum, mandibular midline frenum, the right and left upper and lower buccal frenums and the lingual frenum. The primary function of the frenum is to keep a balance between the growing bones, the tongue and the lip musculature during the development of the foetus and limit the movement of the muscular tissues like the lip, tongue and cheeks [1]. Abnormal frenal attachment may affect the movement of the above-mentioned structures, which in turn may have an effect on the position of jaws and arrangement of dentition.

**Figure 1:**
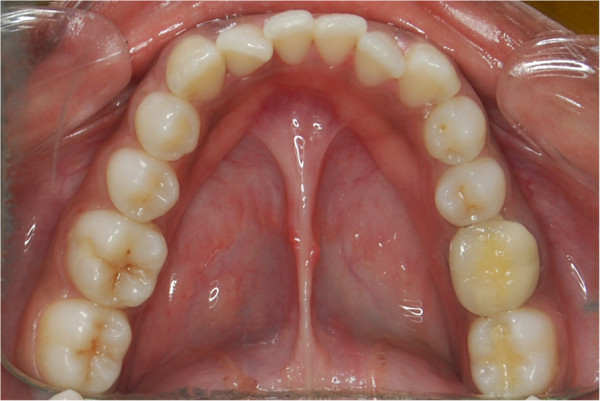
**Ankyloglossia.**

More frequently discussed frenal anomaly in the literature is thick fibrous labial frenum, which causes maxillary midline diastema. Abnormal attachment of lingual frenum, called as ankyloglossia, is a congenital anomaly characterized by short lingual frenum (Figure 1). Ankyloglossia, commonly known as tongue tie, has a prevalence of 4.2% to 10.7% in the population [2]. There is a mild male predilection with a ratio 1.5:1 [3, 4]. Genetic role in the etiology of the ankyloglossia has been discussed by [5], who proposed the possible involvement of human G-protein coupled receptor gene (Lgr5). Ankyloglossia is also inherited as a familial condition either isolated or associated with other anomalies like X-linked cleft palate mutation of gene encoding transcription factor TBX22 [5], Kindler's syndrome [6] and Vander Woude syndrome [7]. It is inherited as an autosomal dominant condition with male to male transmission. The tongue exerts an outward pressure on the teeth, which is counteracted by the constricting effect of the buccal musculature. Equilibrium between these two groups of musculature is necessary for maintenance of arch widths [8]. Hence, altered position of the tongue can also affect the position of the mandible [9]. Ankyloglossia with varying degrees of restricted tongue mobility can cause feeding difficulties during infancy and speech problems and can affect the position of the mandible and the alignment of teeth leading to malocclusion. Due to the restricted mobility, the tongue cannot be lifted upward and subsequently may lead to tongue thrust and open bite [1]. The purpose of the study was to assess certain skeletal and dental characteristics seen in subjects with ankyloglossia and discuss the role of ankyloglossia in etiology of certain malocclusion.

## Methods

This is a case–control study. The study included two groups (groups 1 and 2). Group 1 included 57 subjects diagnosed with ankyloglossia, from patients referred to the Department of Orthodontics, Faculty of Dental Sciences, Sri Ramachandra University, Chennai, India for orthodontic treatment. The sample size was calculated based on the formula which calculates the sample based on the prevalence of the condition.

where *N* is the sample size, *t* is the confidence interval at 95 % (standard value 1.96) and *m* is the margin of error at 5 % level (standard value 0.05).

The diagnosis of ankyloglossia was based on visual inspection and the criteria put forward by [10]. The mean age was 19.02 years. Subjects with only permanent dentition and without any history of previous orthodontic treatment were included in the study. Subjects in primary dentition and mixed dentition and subjects with neuromuscular problems and severe skeletal problems with asymmetry were excluded from the study. Subjects with missing teeth other than third molars were also excluded from the study. The group 2 which comprised controls had 60 subjects selected consecutively from patients, who reported to the department for orthodontic treatment and had class I skeletal base with malocclusion. The principle behind including subjects with malocclusion in group 2 was to find out whether ankylglossia can be attributed exclusively to changes in the parameters in group 1, as parameters such as crowding, open bite and arch constriction can even occur without ankyloglossia. The criteria for class I skeletal base was based on ANB angle 2° ± 2° [11]. The inclusion criteria and exclusion criteria for the control group was the same as that of the experimental group. The proposal was approved by the Institutional Ethical Committee of Sri Ramachandra University. An informed consent was obtained from the subjects participating in the study.

### Measurement of ankyloglossia

**Figure 2:**
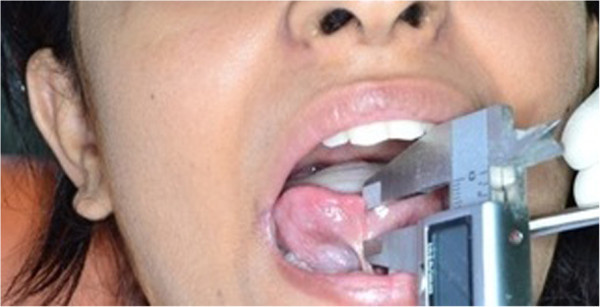
**Measurement of ankyloglossia with digital calliper.**

The measurement of ankyloglossia was based on the classification and grading system given by [10]. It was done using a digital vernier calliper which could make a minimum measurement of 0.01 mm. The distance between the tip of the tongue and the point of attachment of the lingual frenum was measured in millimetres (Figure 2). Clinically acceptable normal range for free tongue is 16 mm. The various grades of ankyloglossia are as follows:

- Class I: mild ankyloglossia (12 to 16 mm)
- Class II: moderate ankyloglossia (7 to 12 mm)
- Class III: severe ankyloglossia (3 to 7 mm)
- Class IV: complete ankyloglossia (<3 mm)

As the tongue is a more flexible organ, a dental instrument is placed at the base of the tongue where the frenum gets attached to the tongue for stabilization. Each measurement was done by the principal investigator. The dental characteristics were measured using study models prepared as part of the diagnostic record. The dental characteristics recorded include maxillary intercanine and intermolar widths, mandibular intercanine and intermolar widths, maxillary and mandibular tooth size-arch length discrepancy and overbite. The measurements were made with the same digital vernier calliper. The maxillary and the mandibular intercanine widths are measured as the distance in millimetres between the cusp tips of the right and the left canine [12]. Maxillary intermolar width was measured as the distance between the mesial fossa of the right and left maxillary first permanent molars, and the mandibular intermolar width was measured as the distance between the central fossa of the right and left mandibular permanent first molars [12]. Maxillary and mandibular tooth size-arch length discrepancy (TSALD) is measured using arch perimeter and Carey's analysis [13]. The overbite was measured using a metal ruler.

### Skeletal measurements

Skeletal measurements were done using a lateral cephalogram. Lateral cephalograms were traced using a 0.03-in. acetate paper. Parameters such as SNA, SNB, ANB, Go-Gn-SN angle from Steiner's analysis [11] and Frankforts mandibular plane angle [14] were recorded. According to the ANB value, the skeletal base was categorized into class I (ANB ± 2°, class II (ANB > 4°) and class III (ANB < 0°).

All the linear and angular measurements were repeated for 14 days by the principal investigator. The method error of measurements was calculated using Dahlberg's formula [15]:

where *d* is the difference between two measurements made from the same parameter and *n* is the number of subjects.

The error of measurement for ankyloglossia was 0.22 mm. The linear measurements ranged from 0.12 to 0.34 mm and from 0.34° to 0.41° for angular measurements.

The various parameters were statistically analysed using SPSS software. Independent *t* test was used to compare the means in both group 1 and group 2. ANOVA followed by Tukey honestly significant difference were done to compare the means among the various grades of ankyloglossia.

## Results

### Measurements of ankyloglossia

The mean ankyloglossia measurement was 8.32 mm in group 1 and 19.17 mm in group 2 (Table 1). In group 1, 59.6% were males and 40.4% were females, showing a slight male predilection (male to female ratio 1.5:1). In group 1, 37 (64.91%) patients had moderate ankyloglossia, 12 (21.05%) patients had mild ankyloglossia and only 8 (14.03%) subjects had severe ankyloglossia (Table 2). There was not a single subject with very severe ankyloglossia.

**Table 1:** **Measurements of ankyloglossia**

Groups	Sex (%)		Mean measurement on the tongue (mm)
	Male	Female	
1	59.65	40.35	8.32
2	53.33	46.66	19.17

**Table 2:** **Grade of ankyloglossia**

Mild	Moderate	Severe
12 (21.05%)	37 (64.91%)	8 (14.03%)

Number of subjects in group 1 and percentage.

### Measurements in lateral cephalogram

In group 1, 70.16% of the subjects belonged to class I skeletal base, 26.31% of the subjects belonged to class II skeletal base and 3.5% belonged to class III skeletal base. In independent *t* test, none of the cephalometric parameters were statistically significant (Table 3). ANOVA (Table 4) showed that none of the parameters were statistically significant except Go-Gn-SN angle (*P* = 0.047) and overbite (*P* = 0.001). In the *post hoc* test (Table 5), Go-Gn-SN angle was statistically significant (*P* = 0.038) between moderate (class II) and severe ankyloglossia (class III). The mean Go-Gn-SN angle in mild ankyloglossia (32.38° ± 3.114°) was greater than the mean angle in severe ankyloglossia (28.75° ± 4.634°).

**Table 3:** **Independent** ***t*** **test for intergroup comparison of means between group 1 and group 2**

Parameters (deg)	Groups	Mean	Standard deviation	***P*** value
Go-Gn-SN angle	1	31.84	4.225	0.372
	2	31.08	3.758	
FMA	1	26.75	4.563	0.200
	2	25.74	3.070

**Table 4:** **ANOVA for comparison of means of parameters among the different grades of ankyloglossia**

Parameters (deg)	Grades of ankyloglossia	Mean	Standard deviation	***P*** value
Go-Gn-SN angle	Grade 1	32.38	3.114	0.047^a^
	Grade 2	32.73	5.042	
	Grade 3	28.75	4.634	
FMA	Grade 1	26.13	3.399	0.142
	Grade 2	27.51	4.181	
	Grade 3	24.83	4.345	

^a^The mean difference is significant at the 0.05 level.

**Table 5:** **Tukey HSD for comparison of means among the different grades of ankyloglossia individually**

Parameters (deg)	Grades of ankyloglossia	Intergroup comparison between grades of ankyloglossia	Significance
Go-Gn-SN angle	Grade 1	Grade 2	0.980
		Grade 3	0.225
	Grade 2	Grade 1	0.980
		Grade 3	0.038^a^
	Grade 3	Grade 1	0.225
		Grade 2	0.038^a^
FMA	Grade 1	Grade 2	0.665
		Grade 3	0.772
	Grade 2	Grade 1	0.665
		Grade 3	0.133
	Grade3	Grade 1	0.772
		Grade 3	0.133

^a^The mean difference is significant at the 0.05 level.

### Measurements on dental casts

In independent *t* test (Table 6), the statistically significant variables were mean maxillary intercanine width (33.005 ± 3.269 mm, *P* = 0.000), mean mandibular intercanine width (26.584 ± 2.363 mm, *P* = 0.005), mean maxillary intermolar width (42.8074 ± 2.50223 mm, *P* = 0.000) and maxillary tooth size-arch length discrepancy (3.60 ± 3.2.983 mm, *P* = 0.005). The mean maxillary and mandibular intercanine widths and the maxillary intermolar width in group 1 were lesser in magnitude when compared to group 2.

**Table 6:** **Independent** ***t*** **test for intergroup comparison of means between groups 1 and 2**

Parameters (mm)	Groups	Mean	Standard deviation	***P*** value
Maxillary intercanine width	1	33.0084	3.983	0.000^a^
	2	35.4022	2.937	
Mandibular intercanine width	1	26.5837	2.858	0.005^a^
	2	27.9598	1.621	
Maxillary intermolar width	1	42.8074	4.938	0.000^a^
	2	48.3738	4.200	
Mandibular intermolar width	1	39.8951	4.197	0.122
	2	41.0918	4.275	
Maxillary tooth size-arch length discrepancy	1	3.60	3.26950	0.010^a^
	2	5.05	3.17573	
Mandibular tooth size-arch length discrepancy	1	3.49	2.36347	0.057
	2	4.40	2.74916	
Overbite	1	2.75	2.157	0.468
	2	2.98	1.282	

^a^The mean difference is significant at the 0.05 level.

In ANOVA, there was no statistical significance in the measurements made on the dental casts except for overbite (*P* = 0.001, Table 7). In the *post hoc* test (Table 8), mean overbite was statistically significant between mild and severe ankyloglossia (*P* = 0.001) and also between moderate and severe ankyloglossia (*P* = 0.016). The mean overbite was reduced in mild (2.13 ± 1.642 mm) ankyloglossia, and it was increased in severe (4.67 ± 1.435 mm) ankyloglossia.

**Table 7:** **ANOVA for comparison of means of parameters among different grades of ankyloglossia**

Parameters (mm)	Grades of ankyloglossia	Mean	Standard deviation	***P*** value
Maxillary intercanine width	Grade 1	34.6013	3.09882	0.324
	Grade 2	32.6724	3.01512	
	Grade 3	32.9825	4.04118	
Mandibular intercanine width	Grade 1	26.1613	2.29260	0.624
	Grade 2	26.8103	2.26657	
	Grade 3	26.1667	2.79068	
Maxillary intermolar width	Grade 1	43.2563	3.69900	0.228
	Grade 2	43.0678	2.30516	
	Grade 3	41.7050	2.01913	
Mandibular intermolar width	Grade 1	38.5275	3.92119	0.422
	Grade 2	40.3730	3.98773	
	Grade 3	39.3333	3.79793	
Maxillary tooth size-arch length discrepancy	Grade 1	1.88	4.155	0.177
	Grade 2	3.57	3.637	
	Grade 3	4.83	1.899	
Maxillary tooth size-arch length discrepancy	Grade 1	2.50	2.000	0.481
	Grade 2	3.49	3.288	
	Grade 3	4.17	2.480	
Overbite	Grade 1	2.13	1.642	0.001^a^
	Grade 2	2.26	2.127	
	Grade 3	4.67	1.435	

^a^The mean difference is significant at the 0.05 level.

**Table 8:** **Tukey HSD for comparison of means among the different grades of ankyloglossia individually**

Parameters (mm)	Grades of ankyloglossia	Intergroup comparison between grades of ankyloglossia	Significance
Maxillary intercanine width	Grade 1	Grade 2	0.291
		Grade 3	0.526
	Grade 2	Grade 1	0.291
		Grade 3	0.956
	Grade3	Grade 1	0.526
		Grade 2	0.956
Mandibular intercanine width	Grade 1	Grade 2	0.766
		Grade 3	1.000
	Grade 2	Grade 1	0.766
		Grade 3	0.697
	Grade 3	Grade 1	1.000
		Grade 2	0.697
Maxillary intermolar width	Grade 1	Grade 2	0.979
		Grade3	0.363
	Grade 2	Grade 1	0.979
		Grade 3	0.232
	Grade 3	Grade 1	0.363
		Grade 2	0.232
Mandibular intermolar width	Grade 1	Grade 2	0.458
		Grade 3	0.896
	Grade 2	Grade 1	0.458
		Grade 3	0.708
	Grade 3	Grade 1	0.896
		Grade 2	0.708
Maxillary tooth size-arch length discrepancy	Grade 1	Grade 2	0.421
		Grade 3	0.152
	Grade 2	Grade 1	0.421
		Grade 3	0.512
	Grade 3	Grade 1	0.152
		Grade 2	0.512
Mandibular tooth size-arch length discrepancy	Grade 1	Grade 2	0.677
		Grade 3	0.448
	Grade 2	Grade 1	0.677
		Grade 3	0.774
	Grade 3	Grade 1	0.448
		Grade 2	0.774
Overbite	Grade 1	Grade 2	0.984
		Grade 3	0.016^a^
	Grade 2	Grade 1	0.984
		Grade 3	0.001^a^
	Grade 3	Grade 1	0.016^a^
		Grade 2	0.001^a^

^a^The mean difference is significant at the 0.05 level.

## Discussion

According to Melvin Moss, the growth of soft tissues has a strong influence over the growth of hard tissues [16]. The tongue is also a soft tissue component which can affect the growth of the maxilla and mandible [9].

The equilibrium between the tongue and buccinator muscle is responsible for the development of normal arch width of the maxillary and mandibular arches [8]. The size, position, structure and function of the tongue also have a potential role in the etiology of malocclusion. Tongue tie or ankyloglossia, which significantly affects the function of the tongue, in turn influences the development of the maxilla and mandible and also the arrangement of teeth. Most of the studies on ankyloglossia were mainly about feeding and speech difficulties in children.

This study used Kortlow's classification of ankyloglossia to diagnose and grade ankyloglossia. Many methods had been put forward to assess tongue tie [10, 17–22]. Most of the above-mentioned methods involved more than one measurement and were too cumbersome. Kotlow's classification was used in this study, as it was very simple, involved only one measurement and easy to perform. This method used a calliper to measure the distance between the point of attachment of the frenum on the ventral surface of the tongue and the tip of the tongue.

Results in this study showed that ankyloglossia was more common in males compared to females (male/female ratio 1.5:1) (Table 1). This was similar to the findings by [3, 4, 19]. The results depicted that moderate ankyloglossia was more common than mild and severe ankyloglossia (Table 2). There was no case of very severe ankyloglossia in the study sample, as the mean age in this group included in this study is 19.02 years and a case of a very severe ankyloglossia would have been diagnosed early in childhood and surgical correction would have been performed because of the feeding and speech difficulties imposed by it. Only some authors had worked on the relationship between malocclusion and ankyloglossia. In this study, majority of group 1 subject belonged to class I skeletal base followed by class II and class III skeletal bases, respectively (Table 9). This was contradictory to other authors [23–25] who reported that ankyloglossia was more common in class III skeletal malocclusion [26, 27] demonstrating no relation between the short lingual frenum and any dental or orthodontic anomalies. It was hypothesized that protrusive chin in ankyloglossia may be due to low tongue posture, which causes the mandible to grow more forward [9, 22]. However, in this study, only two subjects with ankyloglossia belonged to class III malocclusion. The reason could be that ankyloglossia may be an associative factor adding to the genetic component in the etiology of class III malocclusion rather than the sole etiologic factor. The mean Go-Gn-SN plane angle was greater and statistically significant in mild ankyloglossia when compared to severe ankyloglossia (Table 5) suggesting a more backward rotation of mandible. Although the mean Frankfurt mandibular plane angle was not statistically significant among the various grades of ankyloglossia, the value was greater in mild ankyloglossia than in severe ankyloglossia similar to Go-Gn-SN angle, indicating that the position of the tongue is altered in ankyloglossia which in turn can affect mandibular rotation (Table 5).

**Table 9:** **Measurements on lateral cephalogram**

Skeletal base		
**Class I**	**Class II**	**Class III**
40 (70.16%)	15 (26.31%)	2 (3.5%)

Number of subjects in group 1 and percentage.

In this study, there was a significant reduction in maxillary intermolar width, maxillary intercanine width and mandibular intercanine width in group 1 when compared to group 2. However, there was no change in mandibular intermolar width (Table 6). The authors in [9] stated that ankyloglossia limits the upward movement of the tongue, thus preventing the formation of lip seal during swallowing, leading to tongue thrusting, which in turn can cause open bite. Also, the upward movement of the tongue is necessary for the creation of normal width of the hard palate. Inability of the tongue to lift upward results in unrestricted buccinator muscle activity, resulting in constriction of maxillary arch, which could be the reason for reduction in maxillary intermolar width and intercanine width in group 1. Reduction of mandibular intercanine width can be due to pull of the short lingual frenum resulting in constriction of mandibular anterior region. There was no significant difference in maxillary and mandibular intermolar widths and maxillary and mandibular intercanine widths between mild, moderate and severe ankyloglossia.

TSALD in maxillary and mandibular arches was present in both group 1 and group 2 and was statistically significant in the maxillary arch (Table 6). The mandibular and maxillary anterior crowding could be due to arch constriction seen in maxillary and mandibular regions. However, the magnitude of mean TSALD was greater in group 2 when compared to group 1. The reasons for increased TSALD in group 2 could be due to the severity of the malocclusions included in that group.

The overbite was not statistically significant in the independent *t* test (Table 6), but in ANOVA, it was statistically significant among mild, moderate and severe ankyloglossia (Table 7). In *post hoc* comparison, the mean overbite was statistically significant between mild and severe ankyloglossia and moderate and severe ankyloglossia. The mean overbite was greatest in severe ankyloglossia and least in mild ankyloglossia (Table 8). The authors in [9] demonstrated that maxillary arch constriction, maxillary protrusion, crowding and open bite were more common in subjects with ankyloglossia compared to deep bite and spacing. Although the overbite was reduced in mild and moderate ankyloglossia, there was no open bite among those subjects. Though there was a statistical significance in the mean overbite values between mild and severe and moderate and severe ankyloglossia, the difference in the mean values was not greater for it to be clinically significant. This was contrary to the previous hypothesis that ankyloglossia can result in tongue thrust leading to open bite. This showed that restricted mobility of the tongue can cause tongue thrust but not severe enough to cause an open bite. Also, increased mandibular plane angle in mild ankyloglossia could be related to decreased overbite in them. Overbite reduction was not seen in severe ankyloglossia, because the restriction of mobility would be greater and hence less chance to develop a tongue thrust habit to decrease the overbite.

This study has highlighted that ankyloglossia could be related to maxillary arch constriction both in the anterior and posterior regions and constriction of arch in the mandibular anterior region. Ankyloglossia also affects the overbite and the mandibular plane angle. Hence, subjects with ankyloglossia can be considered for surgical correction before orthodontic treatment, as improper tongue function due to ankyloglossia can affect the facial growth and also the outcome of treatment [9].

There were certain limitations in this study. A better cause and effect relationship between ankyloglossia and malocclusion can be brought out with a longitudinal study or a twin study. Also, the present study did not consider the functional aspects of ankyloglossia as it was not under the preview of the study. The various movements of tongue like lateralization, lift, extension, spread, cupping and peristalsis movements should be recorded and related to the skeletal and dental changes in ankyloglossia for future research.

## Conclusions

The present study investigated certain skeletal and dental characteristics in subjects with ankyloglossia. There was a slight male predilection in subjects with ankyloglossia. Moderate ankyloglossia was more common than mild and severe ankyloglossia. Ankyloglossia was more common in class I skeletal base followed by class II skeletal base and class III skeletal base. Maxillary intermolar width and maxillary intercanine width are significantly reduced in subjects with ankyloglossia suggesting maxillary constriction. Overbite and mandibular plane angle changed with the severity of ankyloglossia.

## Authors' information

BS is a senior lecturer and ABC is a professor and head of the Department of Orthodontics, Sri Ramachandra University, Porur, Chennai 600116, India.
